# Seasonal patterns and space‐time clustering of porcine reproductive and respiratory syndrome (PRRS) cases from 2008 to 2016 in Vietnam

**DOI:** 10.1111/tbed.13122

**Published:** 2019-01-30

**Authors:** Hu Suk Lee, Thanh Long Pham, Tien Ngoc Nguyen, Mihye Lee, Barbara Wieland

**Affiliations:** ^1^ International Livestock Research Institute (ILRI) Hanoi Vietnam; ^2^ Epidemiology Division Department of Animal Health Hanoi Vietnam; ^3^ Medical Microbiology Department The Royal Bournemouth Hospital Bournemouth UK; ^4^ International Livestock Research Institute (ILRI) Addis Ababa Ethiopia

**Keywords:** transmission, veterinary epidemiology, virus

## Abstract

Porcine reproductive and respiratory syndrome (PRRS) is an important disease in pig production and is endemic in Vietnam. No nationwide studies have been carried out to understand the spread of PRRS in Vietnam. The main objective of this study was to identify the seasonal patterns and space‐time clusters of PRRS from 2008 to 2016 using national surveillance data in Vietnam. A total of 614,219 cases were reported during the period. There was a seasonal pattern with single peak by region (except North Central Coast, showing double peaks in March and June). The seasonal plots from the Northern regions showed a higher peak between March and April, whereas the four regions from Southern part displayed a higher peak between June and August. Overall, outbreaks from the northern part of Vietnam tended to occur 3–4 months earlier than the southern part. When the spatial window was set at 50%, space‐time cluster analysis found that the first cluster occurred in the Red River Delta (RRD) (radius: 82.17 km; ratios: 5.5; period: Mar–May/2010) and the second (radius: 50.8 km; ratios: 10.61; period: Aug–Oct/2011) in the Mekong River Delta (MRD) region. Four other clusters were observed in the central and Southern parts. Our findings might provide better insight into the distribution of clusters and temporal patterns of PRRS in Vietnam. This study may provide policy makers with valuable information on the hotspot areas and timing of outbreaks. Also, it identifies when and where national control program could be implemented more efficiently by targeting resources for the prevention and control of PRRS.

## 1 INTRODUCTION

Porcine reproductive and respiratory syndrome (PRRS) virus has been recognized as one of the key pig diseases caused by a small RNV virus, which is a member of the order *Nidovirales* and *Arteriviridae* family (Dea, Gagnon, Mardassi, Pirzadeh, & Rogan, 2000; Meulenberg et al., 1993; OIE, 2008; Thiel, G. Meyers, N. Tautz, & G. Unger, 1993). Currently, two genotypes of virus have been described, the European 1 and North American 2 strains, which roughly share 60% nucleotide identity (Murtaugh, Elam, & Kakach, 1995; Nelsen, Murtaugh, & Faaberg, 1999). PRRS is World Organisation for Animal Health (OIE)‐listed disease given its socioeconomic impact (OIE, 2017). The disease causes abortion, stillborn or mummified piglets and high mortality in piglets, and clinical signs may include fever, sneezing, coughing, pneumonia, pyrexia and anorexia in all age groups (Wensvoort, 1993; Zimmerman, Yoon, Wills et al., 1997; Rossow, 1998; Stadejek et al., 2002; OIE, 2008). This virus was first reported in 1987 in the United States of America, and discovered in the 1990s in Europe and since then it has spread round the world (Albina, 1997; Done, Paton, & White, 1996; Wensvoort et al., 1991). In Asia, the virus was first detected in the early 1990s (Murakami et al., 1994; Shimizu et al., 1994). In 2007, a new strain [referred to as ‘highly pathogenic’ (HP) PRRS] emerged in China and Vietnam which is characterized by high morbidity and high mortality rates and spread to other Asian countries which resulted in huge economic losses (Tian et al., 2007; An, Tian, Leng, Peng, & Tong, 2011; Dietze, 2011).

In Vietnam, it has been suggested that PRRS virus may enter through imported pigs from the USA in late 1990s (Nguyen, Vuong, & Vo, 2015). HP‐PRRS virus was first reported in Hai Duong province in 2007 and then detected across the country, which is a partial deletion in the nsp2 compared to the North American genome type (type 2) which is present in Vietnam (Feng et al., 2008; Giang, Lan, Nam, Hirai, & Yamaguchi, 2016; Tian et al., 2007). According to the report from the Department of Animal Health (2009) (N.N., 2009), more than 300,000 pig deaths (including culled pigs for controlling purposes) were reported in 26 of the 62 provinces in 2008. In response to the outbreaks, active surveillance was increased to locate PRRS cases, mainly targeting small and medium farms, and all infected pigs were culled to control and eradicate the disease. The government compensated farmers whose pigs were culled. In high‐risk areas, target pigs (mainly sows and boars) were vaccinated as determined by the local authority, and vaccines were also recommended for other types of pigs. Despite its importance, only a few studies have been conducted to assess the epidemiology of PRRS in Vietnam, comprising case reports, cross‐sectional studies assessing seroprevalence and surveys using molecular tools (Thanh, 2009; Van Cuong et al., 2014). Feng et al. (2008) suggested that PRRS virus from the northern part of Vietnam were 99% similar to Chinese virus at the level of nucleic acid sequences (Feng et al., 2008). In addition, a cross‐sectional study in the Mekong Delta region (MDR) found a seroprevalence of 9.3% (37/397) (Kamakawa, Thu, & Yamada, 2006). One study in the south of Vietnam attempted to identify space‐time clusters using household data, but failed to identify consistent clusters when using different methods (Le, Poljak, Deardon, & Dewey, 2012). To date, no nationwide studies have been carried out to assess the occurrence of cases in space and time. Therefore, the main objective of this study was to identify the seasonal patterns and space‐time clusters of PRRS from 2008 to 2016 using national passive surveillance data in Vietnam.

## 2 MATERIALS AND METHODS

### 2.1 Study location and description of data

Vietnam is the easternmost country on the Indochinese peninsula in Southeast Asia and is a long, narrow nation with an estimated population of 92.7 million in 2016 (GSO, 2018). Weather significantly differs from one region to another due to the length of the country, resulting in considerable variations in the distribution of livestock production systems. Vietnam is officially divided into three administrative hierarchies: provinces (total: 63), districts (total: 724) and communes (total: 11, 181). The number of cases and outbreak dates of PPRS from communes from 2008 to 2016 were obtained from the Department of Animal Health (DAH) in the Ministry of Agriculture and Rural Development (MARD). The national surveillance data were collected on a daily/weekly basis by the local sub‐DAH(s) via email or fax and paper if PRRS cases were confirmed. Most case reports were collected from small and medium farms via passive surveillance. Among reported cases, a few cases were confirmed by national laboratories, whereas most cases were diagnosed on clinical grounds only by local animal health workers. The national surveillance data include the locations of outbreaks (commune, district or provincial level), onset of the outbreaks and number of cases. This study was approved by the Hanoi University of Public Health Review Board (No. 186/2018/YTCC‐HD3), Vietnam.

### 2.2 Data analysis

**Figure 1:**
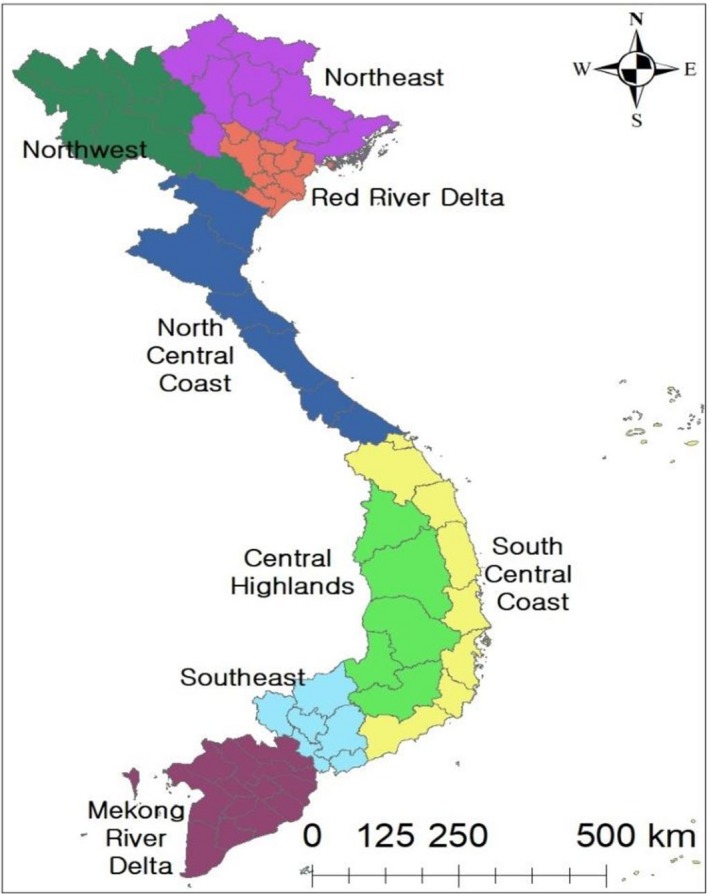
List of regions in Vietnam [Colour figure can be viewed at http://www.wileyonlinelibrary.com]

Vietnam is divided into 63 provinces, and these provinces can be classified into eight ecological zones [(Northeast (NE), Northwest (NW), Red River Delta (RRD), North Central Coast (NCC), Central Highlands (CH), South Central Coast (SCC), Southeast (SE) and Mekong River Delta (MRD)] based on similarities of geographical features and climate conditions (Figure 1). In order to evaluate the temporal patterns of PRRS by each zone, annual pig population for each region was extracted and calculated from the general statistics office of Vietnam (GSO, 2018). Here, we assumed that monthly pig population did not change on a yearly basis for calculating the monthly incidence estimates (per 100,000).

The seasonal‐decomposition procedure based on loess (STL) was conducted by region to evaluate the temporal patterns of PRRS. The STL method is used to decompose time series data into trend, seasonal and remainder on a yearly basis (12 months) (Cleveland, Cleveland, & Terpenning, 1990). In addition, a seasonal cycle subseries plot (SCS) was implemented to assess the monthly variations by each region which supports to visualize patterns both between and within groups.

Basically, this plot shows the average for each month from January to December plotted as a horizontal line during the study period. The vertical lines drawn from the horizontal line display the individual pattern for the same month in each year. Lastly, statistical analysis (using an univariate negative binomial regression (NBR) model) was conducted to evaluate the monthly differences and September was used as a reference (Lee et al., 2014). Incidence rate ratio (IRR) and 95% confidence intervals (CI) were computed by exponentiation of the regression coefficients.

Space‐time cluster analysis was conducted using the SaTScan (version 9.5 free available, http://www.satscan.org), which is commonly used to identify space and space‐time clusters in public health (Kleinman, Abrams, Kulldorff, & Platt, 2005; Kulldorff, 2001). There was a discrepancy of data hierarchy between cases and population as cases were recorded at commune level (total: 11,181), whereas pig population data were available at provincial level (total: 63). It is difficult to justify that entire pigs are at risk at provincial level if outbreaks occur at commune level as province is the largest administrative unit in Vietnam. Therefore, a space‐time permutation model was used to evaluate the space‐time cluster analysis as only reported cases at commune level without data on background pig population at risk for PRRS was available. The number of observed cases in a cluster was compared to what would have expected if the spatial and temporal locations of all cases were independent of each other so that there is no space‐time interaction. For our analysis, the geographical and temporal cluster size were set at maximum of 50% of the population at risk. The cluster was assessed by a maximum likelihood ratio test, and *p*‐value was obtained by Monte‐Carlo simulation with 999 replications of the data set under the null hypothesis. A *p*‐value of <0.05 was considered statistically significant. To support visualization for spatial‐temporal clusters and outbreak locations, maps were generated in ArcGIS version 10.4 ArcMap (ESRI, Redlands, CA, USA). All data were recorded in Microsoft Excel (2013) and analysed using R (version 3.2.2) and STATA (version 14.2, Stata Corp, College Station, TX, USA).

## 3 RESULTS

### 3.1 Seasonal patterns of PRRS cases

**Figure 2:**
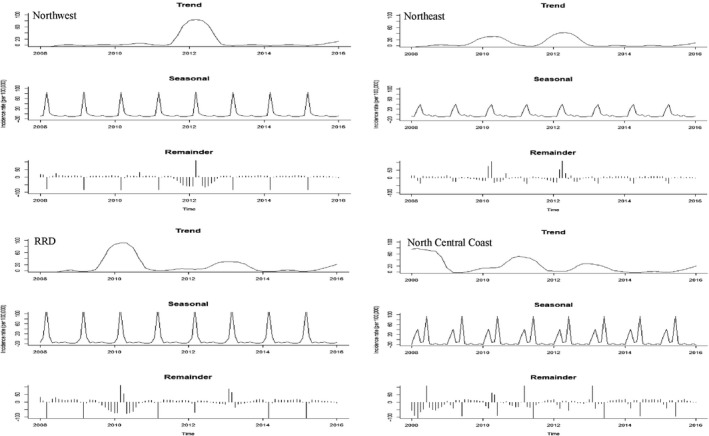
Seasonal‐trend decomposition of the monthly incidence rates (per 100,000) of PRRS in pigs, 2008–2016

**Figure 3:**
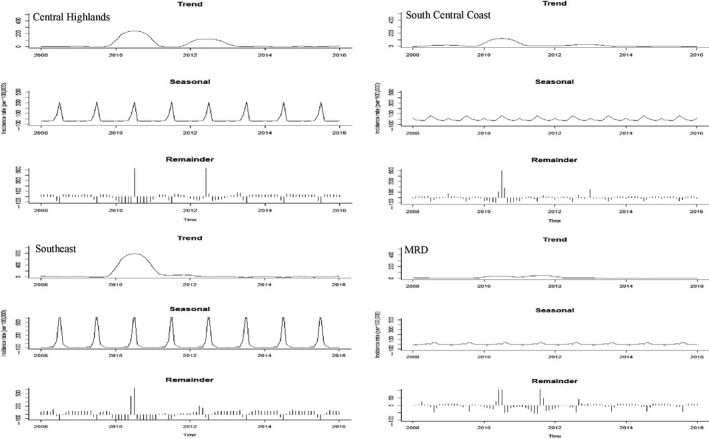
Seasonal‐trend decomposition of the monthly incidence rates (per 100,000) of PRRS in pigs, 2008–2016

**Figure 4:**
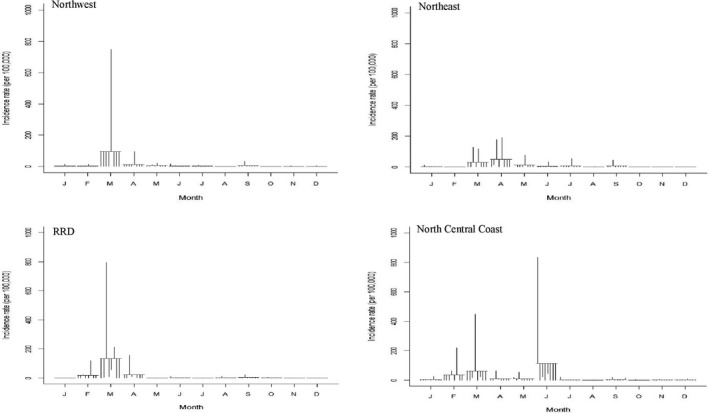
Seasonal cycle subseries plot of the monthly incidence rates (per 100,000) of PRRS in pigs, 2008–2016

**Figure 5:**
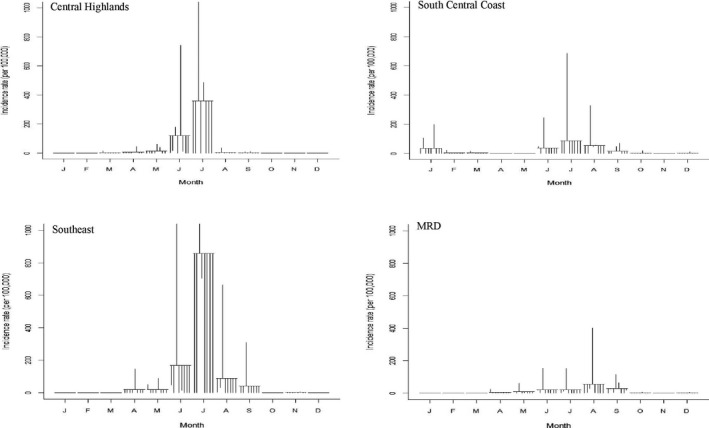
Seasonal cycle subseries plot of the monthly incidence rates (per 100,000) of PRRS in pigs, 2008–2016

A total of 614,219 cases were reported from 2008 to 2016. No cases were reported in 2014, and then few cases were recorded in 2015 and 2016. Overall, the Southern regions (CH, SCC, SE and MRD) had relatively high incidence rates, whereas the NW, NE, NCC and RRD regions had low incidence rates (Figures 2, 3: first plots). STL plots indicated a strong seasonal pattern with single peak by region (except NCC, showing double peaks in March and June) during the study period (Figures 2, 3: second plots). The SCS plots showed relatively high incidence rates from March to April in three regions (NW, NE and RRD) from the northern part, whereas the four other regions (CH, SCC, SE and MRD) from the southern part displayed a higher incidence from June to August (Figures 4, 5). Overall, outbreaks from the northern part of Vietnam tended to occur 3–4 months earlier than the southern part. Univariate NBR analysis showed a significantly increased risk of PRRS in the NW from March to April compared to reference (September) (Table 1). There were significantly higher incidences of disease from April to June, August and October in the NE. In the RRD region, only April showed a significantly increased risk. In the NCC region, most of the months (except from November to January) had significantly higher incidence rates. In the CH and SE regions, significantly higher incidence rates were observed in July and/or August. There were no significantly higher incidences of disease in the SCC and MRD.

**Table 1:** Univariate negative binomial regression (NBR) models for the PRRS by month with incidence rate ratio (IRR) and 95% confidence interval (CI)

Month	Northwest	Northeast	Red River Delta	North Central Coast	Central Highlands	South Central Coast	Southeast	Mekong River Delta
Jan	0.39 (0.14–1.03)	<0.0001	0.01 (0.001–0.34)*	10.94 (0.65–184.44)	Null	0.07 (0.001–0.49)*	Null	0.04 (0.006–0.30)*
Feb	0.45 (0.17–1.21)	16.66 (0.20–1369.03)	Null	15.26 (1.31–177.26)*	Null	1.05 (0.23–4.87)	Null	0.004 (0.001–0.03)*
Mar	23.34 (8.77–62.09)*	<0.0001	6.61 (0.38–114.81)	157.90 (13.60–1832.88)*	Null	0.17 (0.02–1.16)	Null	Null
Apr	2.92 (1.10–7.78)*	126.20 (5.57–2859.86)*	19.96 (1.55–256.46)*	137.62 (14.60–1297.34)*	0.70 (0.05–10.79)	0.08 (0.02–0.38)*	Null	Null
May	0.48 (0.21–1.13)	195.93 (8.65–4439.96)*	4.38 (0.32–59.31)	27.21 (2.69–275.58)*	1.40 (0.15–13.06)	Null	0.63 (0.04–8.96)	0.14 (0.01–1.31)
Jun	0.30 (0.13–0.70)*	44.48 (1.96–1008.15)*	Null	40.51 (3.49–470.38)*	2.82 (0.30–26.31)	Null	0.29 (0.03–2.38)	0.26 (0.04–1.83)
Jul	0.17 (0.06–0.46)*	17.12 (0.76–388.17)	0.58 (0.03–10.02)	334.21 (33.01–3383.76)*	10.49 (1.62–68.01)*	1.00 (0.22–4.66)	1.93 (0.30–12.56)	0.50 (0.08–3.06)
Aug	Null	30.41 (1.34–689.21)*	Null	22.64 (1.35–381.05)*	79.48 (8.52–741.73)*	2.38 (0.51–11.04)	14.81 (1.83–120.10)*	0.63 (0.09–4.52)
Sep	Reference: 1	Reference: 1	Reference: 1	Reference: 1	Reference: 1	Reference: 1	Reference: 1	Reference: 1
Oct	Null	33.86 (1.03–1107.36)*	1.08 (0.06–18.75)	18.21 (1.57–211.58)*	0.54 (0.06–5.09)	0.31 (0.08–1.23)	1.33 (0.09–18.83)	0.52 (0.09–2.86)
Nov	0.07 (0.03–0.19)*	<0.0001	0.41 (0.20–11.03)	3.54 (0.21–59.87)	Null	0.13 (0.02–0.89)*	0.001 (0.001–0.03)*	0.03 (0.01–0.24)*
Dec	0.09 (0.03–0.24)*	<0.0001	Null	6.05 (0.36–102.18)	Null	Null	0.02 (0.001–0.27)*	0.003 (0.001–0.05)*

* Statistically significant at *p* < 0.05.

### 3.2 Space‐time cluster analysis

**Figure 6:**
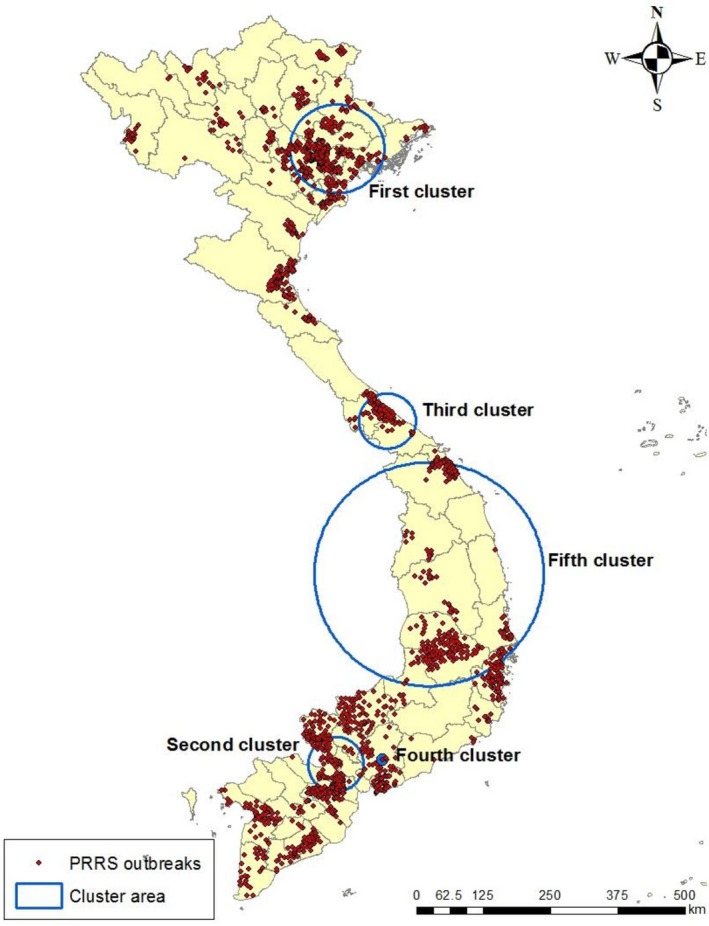
Space–time cluster analysis of PRRS outbreaks from 2008 to 2016 in Vietnam (50% at risk) [Colour figure can be viewed at http://www.wileyonlinelibrary.com]

We explored clustering of cases in space and time, with the spatial window set at 50% of the population at risk. A total of five clusters were identified (Figure 6). The first cluster, observed from March to May in 2010 was in the northern part of Vietnam and other clusters were observed in the central and Southern parts. The first (radius: 82.17 km) and second (radius: 50.8 km) clusters were observed in the RRD and MRD regions which included ratios $(\frac{\text{observed cases}}{\text{expected cases}}),$ 5.5 (76,921/13,849.34) and 10.61 (39,137/3,689.99) respectively (Table 2). The fifth cluster was the largest with a radius of 207.43 km and situated in Dak Lak, Gia Lai, Kon Tum, Quang Nam/Ngai, Binh Dinh and Phu Yen provinces and Cambodia, whereas the smallest size was the fourth cluster (radius: <1 km) in Dong Nai province. The fifth temporal cluster occurred from July 2012 to February 2013. Other temporal clusters were identified between July 2008 and October 2011.

**Table 2:** Space‐time clusters of PRRS in pigs from 2008 to 2016 in Vietnam (space window: 50% at risk)

Cluster no.	Time year/month	Obs/exp = ratio	Radius (km)	*p*‐value
First	Mar/2010–May/2010	76,921/13,849.34 = 5.55	82.17	<0.001
Second	Aug/2011–Oct/2011	39,137/3,689.99 = 10.61	50.8	<0.001
Third	Jul/2008–Aug/2008	30,299/1,814.32 = 16.70	51.01	<0.001
Fourth	Jul/2010–Jul/2010	23,423/1,790.53 = 13.08	0	<0.001
Fifth	Jul/2012–Feb/2013	27,792/4,710.16 = 5.90	207.43	<0.001

## 4 DISCUSSION

This study was the first attempt to evaluate the space‐time clusters and temporal patterns of PRRS using national surveillance data in Vietnam. Not surprisingly, the first cluster was in the RRD which is the main area for intensive pig farming. In fact, most piglets are produced in Thai Binh, Hung Yen, Bac Giang and Ha Nam, and transported to other mountainous areas and to the southern part of Vietnam (Dietze, Pinto, Wainwright, Hamilton, & Khomenko, 2011). Thai Binh is a major collection point from where most of the pigs are transported to other provinces and countries (such as China and Laos). Interestingly, most of the cases were reported in the first half of year (March–April) in the northern part of Vietnam, 3–4 months earlier than in other regions (central and southern part). It is likely that the virus spread from the RRD to other regions, considering pig distribution networks in Vietnam result in movement from the North to the central and southern regions. There is increased animal and human movement before the Tet holiday (Vietnamese New Year, normally in February) which may lead to higher infection rates (Pfeiffer, Minh, Martin, Epprecht, & Otte, 2007). However, as surveillance data showed, higher incidence rates were reported from February to April in the RRD. This delay in reporting may be related to Vietnamese culture. Most of Vietnamese stop working, or at least reduce work to a minimum, about 1 week before Tet, and resume normal work schedules one or even two weeks after the Tet, which may distort national surveillance data. Therefore, it is advised to conduct more in‐depth epidemiological investigations of PRRS and conduct network analysis in the RRD and linkages to other regions.

In Vietnam, vaccination for PRRS was not commonly used at the time the outbreaks reached their peak between 2007 and 2010. At the beginning of the outbreaks, stamping out policies had been implemented to eliminate the disease, but it had been complemented by a vaccination policy in 2009 due to continuous outbreaks. Since then, various PRRS vaccines have been introduced for the pig farmers. In reality, vaccination is mainly used among large‐scale pig farms, but not in small‐scale farms, which account for 70% of the total pig production in Vietnam. The main reason for this is the lack of incentives for pig smallholders to use PRRS vaccine as they can trade pigs without certification of vaccination.

The demand for PRRS vaccine among farmers is high, but farmers are unwilling to pay high prices for vaccinations (Zhang, Kono, Kubota, & Wang, 2015). Therefore, economic reasons are likely the reason for low vaccination rates in Vietnam. As a result, the smallholder sector finds itself in a vicious circle where the PRRS outbreaks continue to occur and biosecurity practices remain very poor, which makes these farms more susceptible for outbreaks. It is necessary to educate smallholders about the importance and economic benefits of regular and systematic vaccination and thorough biosecurity management for control and prevention of PRRS as implementation of an effective culling strategy is difficult in Vietnam (Dietze, 2011). From the government point of view, it is important to provide incentives, such as vaccine subsidy schemes for farmers, to improve immunization coverage rates. Also, characterizing the circulating genotypes in the pig population in a timely manner and match with the correct vaccine is critical, otherwise vaccines are not effective, undermining trust in vaccines of farmers investing in them.

One study has suggested that some avian species (such as chickens and Mallard ducks) are susceptible to PRRS via natural exposure (such as drinking water) and may play a role in the transmission of disease to pigs (Zimmerman, Yoon, Pirtle, Wills, Sanderson, and McGinley, 1997). In 2016, the poultry (mainly chickens and ducks) populations were at 93.6 million and 64.6 million in the RRD and MRD regions respectively (GSO, 2018). Therefore, we cannot ignore the possibility of transmission from poultry to pigs as free‐range chicken and ducks are very common in pig smallholder systems in Vietnam. Further investigations are needed to better understand the role of poultry in PRRS transmission in Vietnam.

A few studies have been conducted to assess the seasonal patterns of PRRS, but results were variable depending on the countries (Arruda, Vilalta, Puig, Perez, & Alba, 2018; Le et al., 2012; Papatsiros, Alexopoulos, & Kyriakis, 2007; Tummaruk et al., 2013). One study suggested that PRRS virus was associated with temperature and/or relatively humidity, whereas temperature had a bigger effect than relatively humidity (Hermann et al., 2007). Recently, it has been suggested that climate factors (temperature and precipitation) and land use were associated with PRRS outbreaks in the United States of America (Alkhamis, Arruda, Morrison, & Perez, 2017). Therefore, it would be worthwhile to evaluate the association between environmental factors (such as temperature, humidity and precipitation) and PRRS outbreaks as weather conditions are significantly different across Vietnam.

In Vietnam, previous studies have suggested the possibility of an increased risk for *S. suis* in humans during the PRRS outbreaks in pigs (Wertheim et al., 2009; Xu et al., 2010). Especially, more human cases with *S. suis* were reported from June and August which was consistent with our results (apart from the northern regions) (Hoa et al., 2013). From a public health point of view, further investigations are required to better understand the association between two pathogens in Vietnam.

Our study had several limitations, mainly related to data quality. First, it was likely that PRRS cases were under‐reported in the remote/mountainous and rural areas due to lack of awareness and professional animal health workers. For example fewer outbreaks were reported in the northern part (mountainous areas) where relatively poorer and more ethnic minorities live. Second, it is possible that reported PRRS cases on clinical grounds may have been caused by other viral or bacterial swine diseases (such as classical swine fever, porcine parvovirus and Aujeszky's disease) since not all cases were confirmed by national laboratories (Dietze et al., 2011; OIE, 2008). Therefore, it is important to improve the national surveillance data by including laboratory confirmation.

We assumed that monthly pig population did not change within the same year which was not realistic. However, the impact of change in pig population on a monthly incidence rate was very limited due to small nominators (cases) versus large denominators (pig population). The STL and SCS analysis are very useful tools to better understand the patterns of time series data (Cleveland et al., 1990). However, SCS plot should be interpreted carefully, because the horizontal lines (average for vertical lines) are highly influenced by large values. In addition, the centroids of each commune were used for our analysis as national surveillance data did not record the coordinates or address for each farm; this may have led to inaccurate results as the negative impact of inaccuracies in spatial event data have been suggested by a number of studies (Burra, Jerrett, Burnett, & Anderson, 2002; DeLuca & Kanaroglou, 2008). Lastly, we assumed that the pig population did not change dramatically over the study period, which is important for a space‐time permutation model as this model cannot distinguish at increase or decrease risk of disease due to a population versus disease increase or decrease. Therefore, identified clusters could have been influenced by pig population at risk if the background population dramatically increases or decreases in one area than in others. However, we confirmed that the total pig population has not changed dramatically from 2008 to 2016, with a slight increase overall (<10%) from 27 to 29 million, minimizing risks of bias in our results^28^.

Our findings might provide better insight into the distribution of clusters and seasonal patterns of PRRS in Vietnam, proving the non‐homogenous distribution of cases and thus warranting more detailed epidemiological investigations. This study may provide policymakers with valuable information on the hotspot areas and timing of outbreaks. It also identifies when and where national surveillance and control programs could be implemented more efficiently for the prevention and control of PRRS. It is important to raise public awareness on vaccination in high‐risk areas during the peak months and seasons to prevent outbreaks and onward transmission.

## CONFLICT OF INTEREST

The authors declare that they have no competing interests.
